# Status Epilepticus Induced Spontaneous Dentate Gyrus Spikes: *In Vivo* Current Source Density Analysis

**DOI:** 10.1371/journal.pone.0132630

**Published:** 2015-07-06

**Authors:** Sean P. Flynn, Sylvain Barrier, Rod C. Scott, Pierre-Pascal Lenck- Santini, Gregory L. Holmes

**Affiliations:** 1 Department of Neurological Sciences, University of Vermont College of Medicine, Burlington, VT, United States of America; 2 University College London, Institute of Child Health, London, United Kingdom; Federal University of Rio Grande do Norte, BRAZIL

## Abstract

The dentate gyrus is considered to function as an inhibitory gate limiting excitatory input to the hippocampus. Following status epilepticus (SE), this gating function is reduced and granule cells become hyper-excitable. Dentate spikes (DS) are large amplitude potentials observed in the dentate gyrus (DG) of normal animals. DS are associated with membrane depolarization of granule cells, increased activity of hilar interneurons and suppression of CA3 and CA1 pyramidal cell firing. Therefore, DS could act as an anti-excitatory mechanism. Because of the altered gating function of the dentate gyrus following SE, we sought to investigate how DS are affected following pilocarpine-induced SE. Two weeks following lithium-pilocarpine SE induction, hippocampal EEG was recorded in male Sprague-Dawley rats with 16-channel silicon probes under urethane anesthesia. Probes were placed dorso-ventrally to encompass either CA1-CA3 or CA1-DG layers. Large amplitude spikes were detected from EEG recordings and subject to current source density analysis. Probe placement was verified histologically to evaluate the anatomical localization of current sinks and the origin of DS. In 9 of 11 pilocarpine-treated animals and two controls, DS were confirmed with large current sinks in the molecular layer of the dentate gyrus. DS frequency was significantly increased in pilocarpine-treated animals compared to controls. Additionally, in pilocarpine-treated animals, DS displayed current sinks in the outer, middle and/or inner molecular layers. However, there was no difference in the frequency of events when comparing between layers. This suggests that following SE, DS can be generated by input from medial and lateral entorhinal cortex, or within the dentate gyrus. DS were associated with an increase in multiunit activity in the granule cell layer, but no change in CA1. These results suggest that following SE there is an increase in DS activity, potentially arising from hyperexcitability along the hippocampal-entorhinal pathway or within the dentate gyrus itself.

## Introduction

Mesial temporal lobe epilepsy is characterized by profound changes in hippocampal and parahippocampal network circuitry resulting in spontaneous recurrent seizures and interictal activity in humans and animal models [1–3]. Under normal conditions granule cells within the dentate gyrus demonstrate low levels of excitability, which effectively gates the flow of information within the hippocampus and limits pyramidal cell excitation [4,5]. Disruption of the gating function of the dentate gyrus has been proposed as a potential mechanism for the generation of ictal and interictal events in humans and animal models [5–7]. Indeed, the dentate gyrus has been shown to be critical in seizure progression facilitating the transition from irregular spiking to bursting [8].

Following status epilepticus (SE), induced either chemically or electrically, dentate granule cells become hyperexcitable [9–11], reducing their ability to properly gate excitatory input from cortical structures. Such hyperexcitability may result from a reduction in hilar mossy cell input onto inhibitory basket cells as well as hilar inhibitory interneuron cell death [12–15]. Synaptic reorganization in the form of mossy fiber sprouting from granule cells and CA3 backpropagating axon collaterals has also been suggested to modify granule cell excitability [16–18]. In addition to modification of local hippocampal networks, SE-induced insults also modify parahippocampal structures. The entorhinal cortex displays considerable cell death following SE in layers III and V [19,20]. Similar to dentate granule cells, layer II entorhinal neurons are hyperexcitable following SE, likely resulting from disinhibition caused by cell death in surrounding layers III and V [21,22]. Since layer II neurons form the main excitatory input to the dentate granule cells [23], it is likely that the entorhinal-hippocampal pathway is critical in the initiation of epileptiform activity following SE.

In the normal brain, the dentate granule cells have been shown to display large amplitude population events, termed DS that act to limit pyramidal cell activity in CA3 and CA1 [24,25]. Current source density (CSD) analysis of these events revealed current sinks in both the outer and middle molecular layers of the dentate gyrus corresponding to input from layer II of the lateral and medial entorhinal cortex [25]. Following kainic acid-induced seizures, there is an increase in inhibition in dentate granule cells observed *in vivo*. This effect is weak and is easily overcome by pathological activity from parahippocampal structures, such as entorhinal cortex, resulting in granule cell population spikes or high amplitude DS not seen under normal conditions [26,27]. It is possible that there are multiple mechanisms through which spontaneous epileptiform activity can be generated within the hippocampus. Indeed, stimulation of the perforant path or commissural system demonstrate that seizure-like activity can be generated in the CA3-CA1 or entorhinal cortex-dentate gyrus circuits independently [28]. In this study, we characterized the initiation of spikes in the dentate gyrus and CA fields following pilocarpine-induced SE. Modification of the physiological properties or initiation site of events like the DS can provide novel insights on SE-induced changes to normal physiological events that are important in the entorhinal-hippocampal pathway.

## Materials and Methods

### Animals

Male Sprague-Dawley (180–200 g) rats were obtained from Charles-River (Saint Constant, QC). All procedures were carried out in accordance with recommendations in the Guide for the Care and Use of Laboratory Animals of the National Institutes of Health. The protocols were approved by the IACUC of the University of Vermont (Protocol Number: 13–054). Rats were housed on a regular 12/12 light cycle and given food and water *ad libitum*.

### Pilocarpine-Induced Status Epileptics

Status epilepticus was induced as previously described [29,30]. Rats were administered lithium chloride (127 mg/kg, i.p.) 18 hrs before SE induction. Thirty minutes before SE induction, rats were separated into individual cages and administered scopolamine methyl bromide (1 mg/kg, i.p.). SE was induced with pilocarpine hydrochloride (12.5 mg/kg, i.p). Doses of pilocarpine were administered every 30 minutes until continuous stage 4/5 seizure activity was observed. Seizure score was determined based on the Racine scale [31]; briefly, stage 4 seizures were defined by bilateral forelimb clonus and rearing; stage 5 seizures were defined as the occurrence of stage 4 behavior plus loss of posture or falling. Stage 5 seizure activity represented a generalized seizure where seizure activity has spread to the whole brain. Animals were maintained in SE for 90 minutes, after which lorazepam (2 mg/kg, i.p) was administered to terminate seizure activity.

### Urethane-Anesthesia Recoding

At least two weeks after SE induction, animals were anesthetized with urethane (1.5 g/kg, i.p) for electrophysiological recording. A craniotomy was prepared over the hippocampal formation (AP: -3.2; ML: -2 to -4) to remove the skull and dura. After craniotomy, silicon probes were placed over the brain at 2.5 mm lateral to bregma to record from the dorsal-ventral axis containing CA1-DG or 3.4 mm lateral to bregma to record from the CA1-CA3 axis. Continuously sampled EEG was recorded across all 16 channels between 1 to 9000Hz (sampling frequency of 30 kHz). All recordings were conducted for at least 20 minutes. DS data were converted to frequency for statistical analysis to allow for comparison of recordings of different lengths of time. In addition, channels on which single unit activity was observed were spliced and single unit activity was recorded in a separate file (30 kHz, 600–6000 Hz). Evaluation of unit activity in concert with stereotaxic positioning of the silicon probe allowed for reliable placement of the probes within the hippocampal formation covering the appropriate cellular layers. All electrodes were coated with DiI prior to placement in the brain to allow for histological verification of probe placement. In some rats, additional recording electrodes, consisting of bundled EEG wires, were placed in the entorhinal cortex (AP: -8.5; ML: -5.3; DV: -5.2) and recorded simultaneously with hippocampal EEG. To further confirm probe placement, stimulating electrodes were placed in the angular bundle (AP: -7.2; ML: -4; DV: -3.2) and the ventral hippocampal commissure (AP: -1.3; ML: 0.1; DV: -5.2).

### Event Detection and CSD Analysis

**Fig 1:**
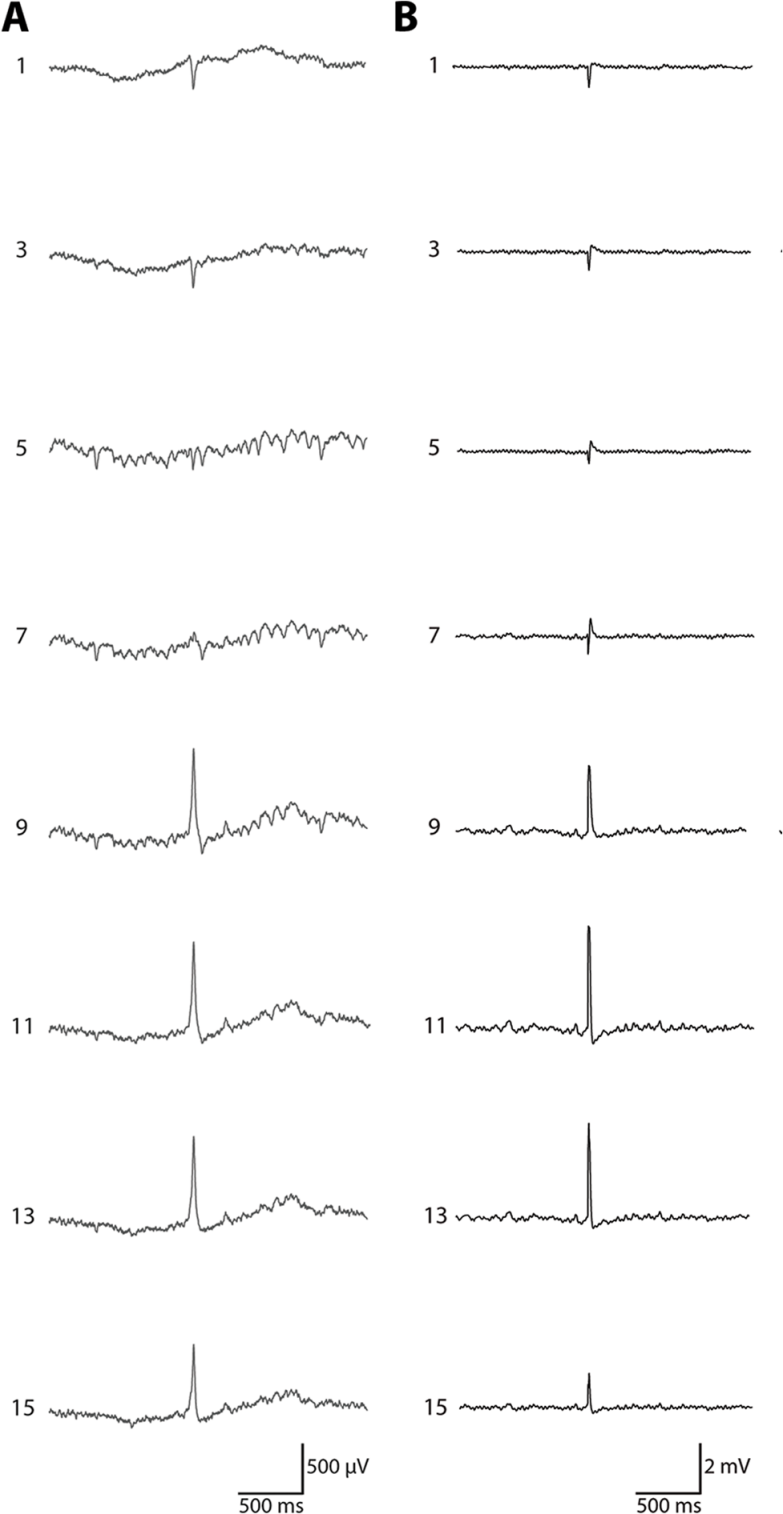
Simultaneous recoding of field activity in the CA1-dentate gyrus axis of dorsal hippocampus. For simplicity, every other trace is shown (16 total recording sites, 100 μm contact intervals). A) A typical dentate spike was depicted in the traces shown, characterized by a large amplitude, highly contoured event with a positive going waveform in the dentate gyrus. The CA1 pyramidal cell layer was located near trace 3 and the dentate granule cell layer was located near trace 9. B) An example recording depicting a characteristic response to a angular bundle stimulation. The silicon probe was located in a similar orientation to the recording shown in panel A. The CA1 pyramidal cell layer was located near trace 3 and the dentate granule cell layer was located between traces 9 and 11.

Spontaneous interictal events were detected from EEG recordings based on threshold values set for each individual recording. All recordings were acquired with the Cheetah32 analog signal recording system interfaced with Cheetah 5 data acquisition software (NeuraLynx, Bozeman, Montana). EEG was reviewed offline utilizing NeuroExplorer software (Nex Technologies, Madison, AL). CSD plots were generated using custom software in MATLAB to localize current sinks and sources across the recoded regions. All recordings across the 16-channels of the silicon probe were processed utilizing the same parameters. EEG traces were filtered with a 1 Hz high pass and 1 kHz low pass filter. Location of the recording channels within the hippocampus was determined based on a combination of three different approaches: 1) the presence of action potentials in recording sites located at the vicinity of a cell layer; 2) histological verification of the probe track identified by the DiI trace under fluorescent microscopy; 3) CSDs induced by the stimulation of the angular bundle in some animals (Fig 1B).

All spike events were defined as a transient EEG event that was sharply contoured, crossing a threshold set as six times the standard deviation of the EEG signal for a given channel and lasting < 50 ms in duration (Fig 1A). DS were defined as having a large amplitude positive going potential with the peak amplitude occurring in the dentate gyrus. In addition, all DS displayed a phase reversal and current sink in the molecular layer or outer molecular layer of the dentate gyrus. These criteria are consistent with previous work [24,25] that defined and characterized these types of events. Interictal spikes were defined as spikes that crossed our threshold, but did not meet the criteria established for DS. For each recording, at least two channels were evaluated for spike activity corresponding to channels in CA1, the dentate gyrus, and CA3. Events did not have to cross the threshold on all channels to be considered an event. All potential events were further evaluated individually and electrical artifacts were removed. Events were then classified based on their width, waveform amplitude and shape, and the recording channel where reversal of the signal was observed. Detection of interictal spikes was verified by manual detection. For individual recordings from both control and pilocarpine-treated animals, EEG across all 16 channels was further reviewed utilizing NeuroExplorer software to verify threshold detected events (Nex Technologies, Madison, AL). CSD plots were generated using custom software in MATLAB. Although some differences in resistance were present in different hippocampal layers, these differences were minimal and were unlikely to modify spatial distribution of current sinks and sources. As such, we assumed isotropy of the extracellular space in CSD analysis.

### Multiunit Activity

To identify and extract single unit activity in cell layers, we filtered recorded EEG traces between 300–6000 Hz and set a threshold of at least 3 times the standard deviation of the filtered signal isolating multiunit activity from the background signal. EEG traces to be analyzed were selected based on two criteria: 1) the presence of single unit activity on filtered EEG traces; 2) histological verification of electrode contact placement near either the CA1 pyramidal cell layer or the granule cell layer in the dentate gyrus. Isolated multiunit activity for CA1 and the dentate gyrus were plotted in perievent time histograms where the peak amplitude of the DS was fixed to time zero. Multiunit activity was plotted in 5 ms bins beginning 100 ms before and ending 100 ms after the peak amplitude of the dentate spike. To assess the significance of event-related activity, a z-score for each 5 ms bin was calculated as previously described [32]. The z-score represents the raw multiunit activity in each bin minus the population mean divided by the standard deviation of the population over the plotted 200 ms. If more than two successive bins show a z-score ≥3 during the analyzed time period the activity was considered to be related to the dentate spike.

### Histology

**Fig 2:**
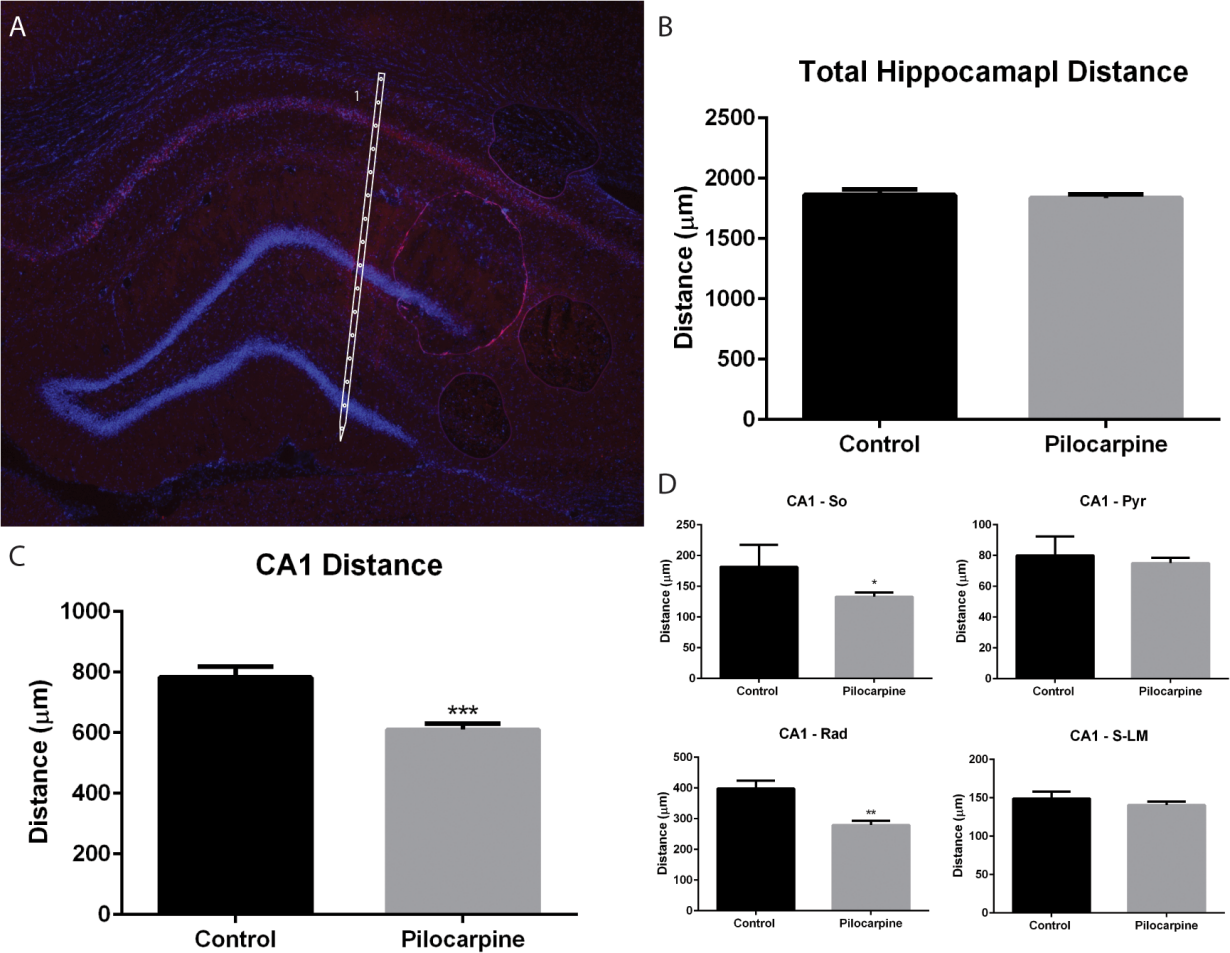
Pilocarpine-induced morphological changes. A) Reconstruction of silicon probe placement within a coronal section of the hippocampus. Scaled silicon probe outlines were superimposed on sections counterstained with DAPI. DiI tracks and multiunit activity guided probe alignment. B) No differences in the distance from stratum oriens in CA1 to the outer molecular layer of the inferior blade of the dentate gyrus were seen between control and pilocarpine animals. C) There was a significant decrease in the distance taken up by area CA1 in pilocarpine-treated animals measured from stratum oriens to the hippocampal fissure. D) Further analysis of CA1 demonstrated a significant decrease in stratum oriens and stratum radiatum in pilocarpine-treated animals, with no change to the pyramidal cell layer or stratum lacunosum-moleculare.

Following recording, animals were transcardially perfused with PSB followed by 4% paraformaldehyde. Brains were removed and post-fixed in 4% PFA for 24 hrs, then moved to 30% sucrose solution until saturated and then frozen and sectioned at 30 μm on a cryostat. Sections were counterstained with DAPI (1:1000) to verify cell layers in relationship to DiI probe tracks (Fig 2A). Additional animals were processed for Timm staining following completion of EEG recording. Timm staining was performed as follows [33]: Rats were perfused with normal saline followed by 200 ml of sodium sulfide, and 200 ml of 4% paraformaldehyde. Brains were removed, post-fixed in 4% PFA for 24 hrs, and placed in 30% sucrose until the brains sank. Coronal sections were taken along the extent of the hippocampus at 40 μm on a freezing cryostat. For Timm staining, sections were developed in a solution of 50% gum arabic (120 ml), 51% citric acid (10 ml), 47% sodium citrate (10 ml), hydroquinone, and silver nitrate for 45 min. After washing slides containing DiI labeling from electrode placements were washed in dH_2_0 and then coverslip with Fluromount G. The remaining slides were dehydrated in alcohol, cleared in methyl salicylate and coversliped with Permount.

## Results

### Pilocarpine-Induced Morphological Changes

Pilocarpine-induced SE has been documented to cause cell death and damage to the hippocampus with a high degree of variability depending on dosing protocols and the duration of SE. Since the anatomical morphology of hippocampal layers in SE rats may influence interpretation of CSD profiles we measured the distance between anatomically defined layers across the dorsal-ventral axis of the hippocampus in both control and SE groups (Fig 2). We observed a significant decrease in the dorsal-ventral distance in the CA1 region in pilocarpine-treated animals (pilocarpine: 610±19.2 μm; control: 783±36.2 μm, p = 0.001, Fig 2C).Further analysis revealed the reduction in CA1 region distance resulted from a specific reduction in size the of CA1 stratum oriens (pilocarpine: 133±7.0 μm; control: 181.6±35.9 μm, p = 0.034) and CA1 stratum radiatum (pilocarpine: 279.4±14.0 μm; control: 398.0±26.4 μm, p = 0.002) in the pilocarpine-treated animals (Fig 2D). Despite the differences in the CA1 region, there was no difference between groups in the total dorsal ventral distance stretching from CA1 stratum oriens to the inferior molecular layer of the dentate gyrus (pilocarpine: 1836±30.4 μm, n = 11; control: 1861±46.2 μm, n = 3, p = 0.74, Fig 2B). Overall, pilocarpine-induced SE decreased the size of CA1 likely due to cell death, and an associated expansion of the molecular layer potentially due to axonal sprouting.

A subset of pilocarpine-treated brains (n = 2) used in our recordings was processed for Timm staining to evaluate changes in the mossy fiber organization. In control animal’s, normal mossy fiber projections were observed arising from granule cells in the dentate gyrus and projecting to the stratum lucidum of CA3. In contrast, in the dentate gyrus of pilocarpine-treated animals, mossy fibers were observed in the granule cell layer and the inner molecular layer. They were also observed in the CA3 pyramidal cell layer. Those changes were indicative of mossy fiber sprouting, where dentate granule cells formed recurrent synaptic contacts onto other dentate granule cells and aberrant excitatory connections to CA3 pyramidal layer [33–35]

### Identification and Classification of Spike Events

**Fig 3:**
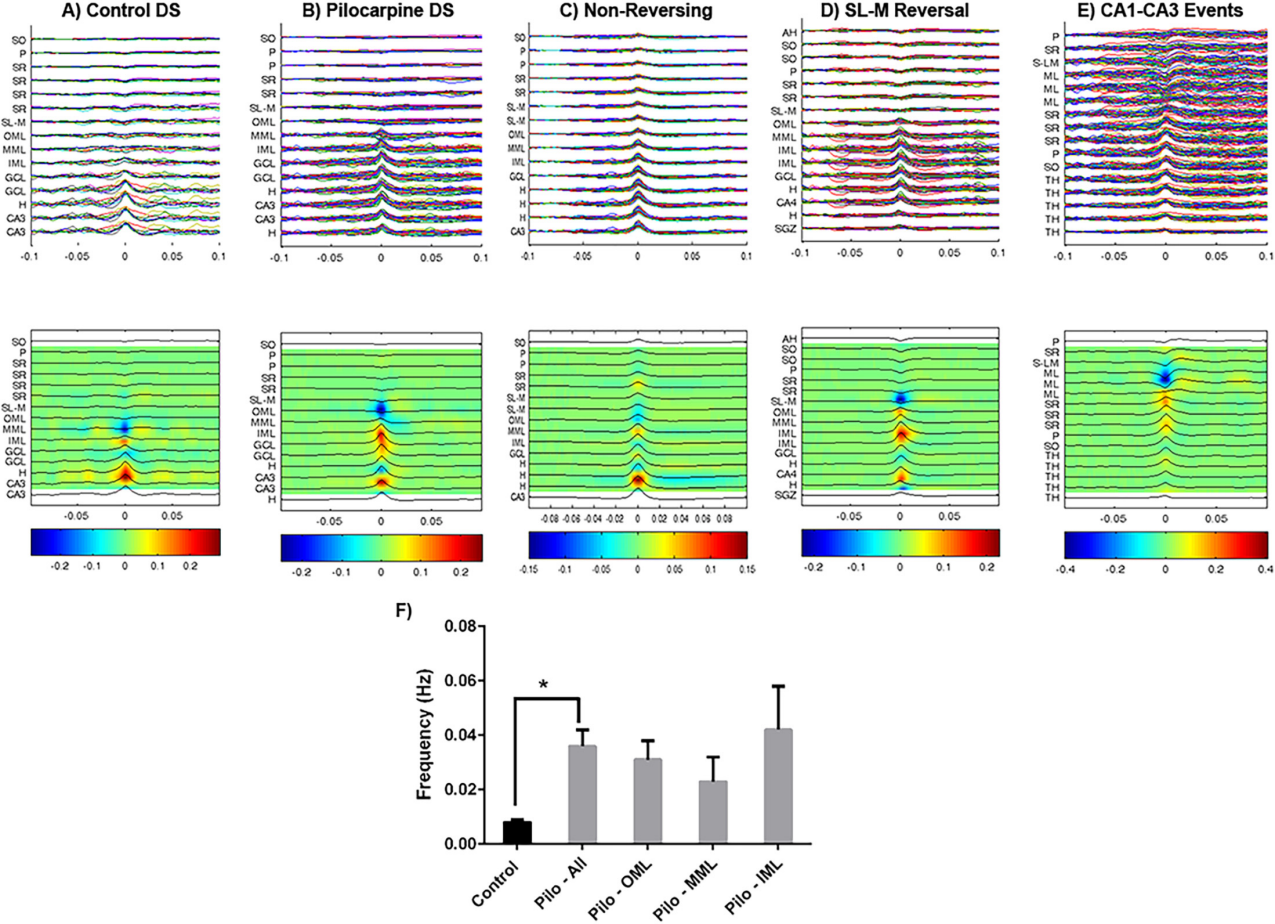
Waveform and CSD plots of identified spikes in control and pilocarpine-treated animals. In the top panel all individual events were overlaid and aligned to the peak spike amplitude. In the bottom panel an average of the EEG traces was shown overlaid on a CSD plot displaying current sinks (blue) and current sources (red). In both panels on the y-axis the anatomical location of each of the 16-laminar (100 μm spacing) EEG traces was shown. A) DS observed in control animals (n = 8 spikes), B) Pilocarpine-induced DS (n = 40), C) Non-Reversing spikes in the CA1-DG plane (n = 58), D) Population events with a reversal in CA1 SL-M (n = 35) E) Events recorded in the CA1-CA3 pane (n = 47). F) Frequency of all DS in control (black) and pilocarpine (grey) treated animals. Frequency of DS in pilocarpine-treated animals was further segregated based on location of current sink in the dentate molecular layer. No significant difference was observed between the frequencies of DS at each layer of the molecular layer. SO–stratum oriens, P–CA1 pyramidal cell layer, SR–stratum radiatum, SL-M–stratum lacunosum moleculare, OML–outer molecular layer of the dentate gyrus, MML–middle molecular layer of the dentate gyrus, IML–inner molecular layer of the dentate gyrus, GCL–granule cell layer, H–hilus, CA3 –CA3 pyramidal cell layer, AH–above hippocampus, ML–molecular layer, TH–thalamus.

Recordings in the hippocampal formation with 16-channel silicon probes were performed in control (n = 3) and pilocarpine-treated (n = 11) animals and analyzed for large amplitude population events. Events were classified based on 1) the orientation of the probe within the hippocampus; and 2) the anatomical location of the major current sink. In control animals, large amplitude (1343.9 ± 88.3 μV) spikes were observed when probes were placed along the dorsal-ventral axis containing CA1 and the dentate gyrus. These events were identical to previously described DS with a positive going potential within the dentate gyrus as well as a phase reversal and current sink in the molecular layer of the dentate gyrus (Fig 3A).

Recording in the same orientation, DS were observed in 9 of the 11 pilocarpine-treated animals. A total of 1,350 events were recorded. The maximum amplitude for DS in both pilocarpine-treated and control animals was observed in the granule cell layer. Similar to control events, pilocarpine DS were characterized by a large amplitude (1497 ± 132.1 μV) positive going population event and a phase reversal and current sink in the molecular layer of the dentate gyrus (Fig 3B). A subset of DS (n = 24) with a current sink in the outer molecular layer displayed an additional, although weaker, current sink in the stratum radiatum of CA1, suggesting the event may have propagated through the hippocampus. Electrode spacing on the silicon probes (100 μm) allowed for three contacts to be located in the molecular layer, allowing for a distinction between activities in the outer, middle, and inner thirds of the layer. As a result, we were able to classify DS based on the site of phase reversal and current sink location within the molecular layer. The majority (n = 866 in 9 animals) of DS displayed a phase reversal and distinct current sink in the outer molecular layer. DS were also observed to have a phase reversal and current sinks in the middle (n = 326 in 6 animals) and inner (n = 158 in 2 animals) molecular layers. Overall, DS in pilocarpine-treated animals occurred with a significantly higher frequency compared to controls (Control: 0.008 ± 0.001 Hz; Pilocarpine: 0.036 ± 0.006 Hz; p < 0.001, Fig 3F). However, there was no difference in the frequency of DS when comparing events that occurred at each third of the molecular layer (Fig 3F, Table 1). Despite their overall increased frequency, there was no difference in the amplitude or width of DS between controls and pilocarpine-treated animals or when comparing DS between layers of the molecular layer (Table 1). Thus, following SE, there is an increase in the frequency of DS, but no change in amplitude or width when compared to control DS.

**Table 1:** Summary of all events and spike types observed in pilocarpine and control treated animals.

Spike Type	# of Events	# of Rats	Frequency (Hz)	Amplitude (μV)	Width (ms)
Control DS	45	2	0.008 ± 0.001	1343.9 ± 88.3	16.4 ± 1.2
Pilocarpine DS–Total	1350	9	0.036 ± 0.006*	1497.0 ± 132.1	17.3 ± 0.6
Pilocarpine DS–OML	866	9	0.031 ± 0.007	1531.6 ± 160.1	17.1 ± 0.7
Pilocarpine DS–MML	326	6	0.023 ± 0.009	1330.8 ± 180.2	18.9 ± 1.1
Pilocarpine DS–IML	158	2	0.042 ± 0.016	1582.9 ± 14.3	15.4 ± 0.3
SL-M Reversal	72	3	0.021 ± 0.012*	1418 ± 335.8	15.9 ± 0.3
Non-Reversing	468	2	0.063 ± 0.019*	950.6 ± 137.3	14.3 ± 1.4
CA1-CA3 Reversing	78	1	0.031	758.9	17.8

* p < 0.001 compared to Control DS

OML–outer molecular layer of the dentate gyrus, MML–middle molecular layer of the dentate gyrus, IML–inner molecular layer of the dentate gyrus

In the two pilocarpine-treated animals that did not display DS, we observed large amplitude events that crossed threshold but showed no phase reversal across the 16 channels recorded (n = 468, Fig 3C). This type of event was never observed in control animals. Although there was no phase reversal, the amplitude of the events varied across the 16 channels, suggesting these events initiated outside the hippocampus and then invaded the hippocampal formation via volume conduction. These non-reversing events occurred at a significantly higher frequency compared to controls (Control: 0.008 ± 0.001 Hz; Non-reversing: 0.063 ± 0.019, p < 0.001), and have significantly smaller amplitude than both control (p < 0.05) and pilocarpine-induced DS (p < 0.01) (Table 1).

A third population of events was observed in pilocarpine-treated animals when recording from the dorsal-ventral axis containing CA1 and the dentate gyrus characterized by a phase reversal and current sink in the stratum lacunosum-moleculare with a corresponding source in in the molecular layer (n = 72, Fig 3D, Table 1). These events were also never observed in control animals. Similar to DS, these events had a significantly higher frequency compared to control animals (Control: 0.008 ± 0.001 Hz; SL-M: 0.021 ± 0.012, p < 0.001), but were not significantly different in frequency from pilocarpine DS. There was no difference in either spike amplitude or spike width compared to controls.

Additional recordings were completed in both control and pilocarpine-treated animals where silicon probes were placed in the dorsal-ventral plane containing CA1-CA3. In this orientation two contacts on the probe were located in the lateral region of the molecular layer of the dentate gyrus. Of those events that crossed threshold, none were observed to initiate in CA1 or CA3. However, in one animal, events were observed with a phase reversal and current sink in the lateral portion of the molecular layer, similar to what had been previously observed with DS (n = 78, Fig 3E). Recordings in control animals (n = 3) with silicon probes displaying similar trajectories through the CA1-CA3 axis did not have any events cross threshold.

All of the pilocarpine-treated rats had spontaneous seizures consisting of bilateral forelimb clonus, with or without rearing. Seizures were not quantified with EEG-video monitoring. No seizures occurred in the hour prior to, or during, the recordings.

### Multiunit Activity

To access how dentate and CA1 neuronal activity was affected by DS, we evaluated changes in multiunit activity before and after each event in the 9 pilocarpine and 2 control animals in which DS were recorded. Perievent histograms were plotted so that time zero on the x-axis corresponds to the peak amplitude of the dentate spike in both control and pilocarpine-treated animals.

**Fig 4:**
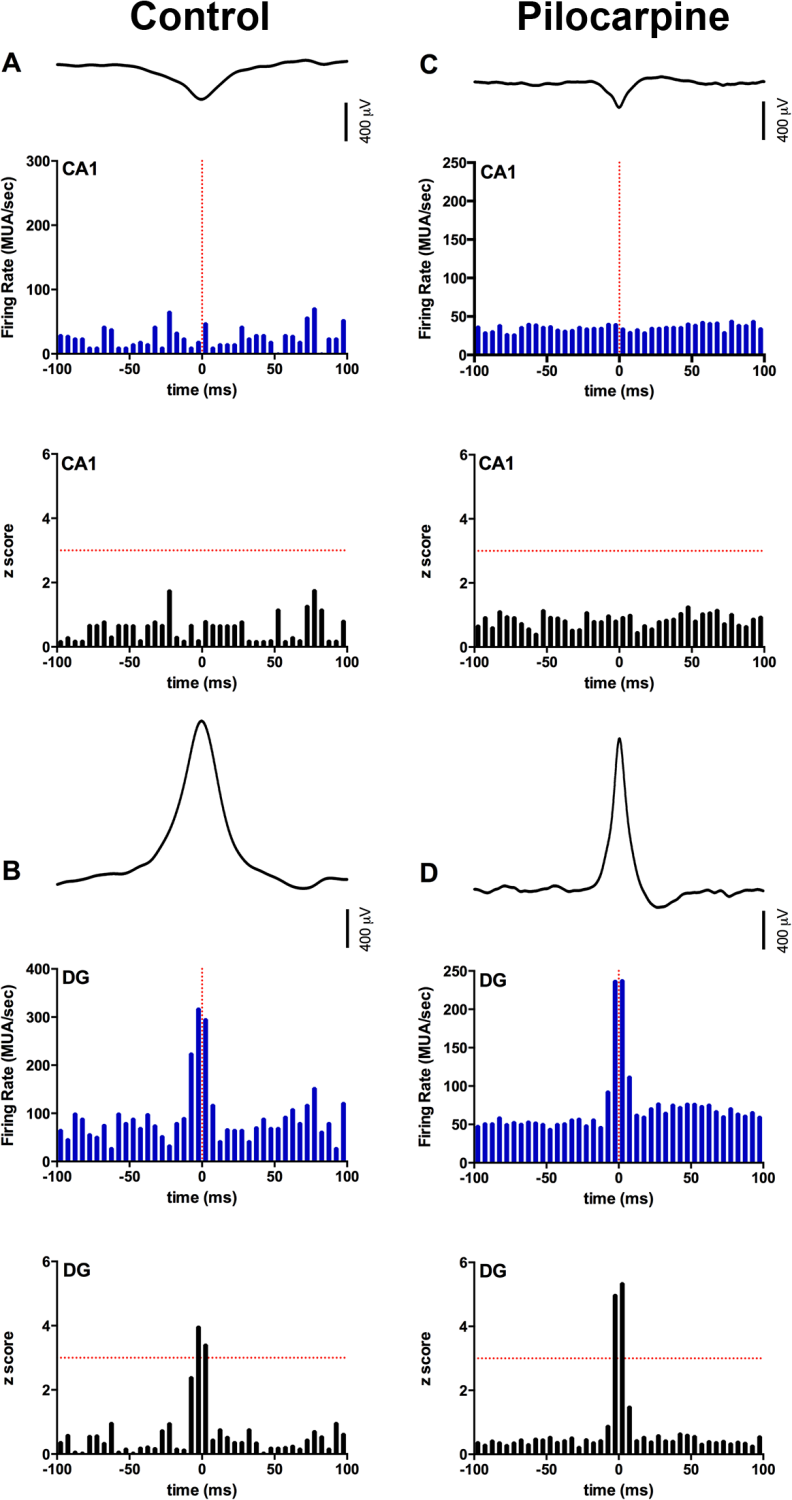
Perievent Time Histogram and z-scores of multiunit activity in the hippocampus during DS. MUA activity and z score of A) CA1 pyramidal cell layer and B) dentate granule cell layer in control animals. MUA activity and z score of C) CA1 pyramidal cell layer and D) dentate granule cell layer in pilocarpine-treated animals. The peak amplitude of DS was locked to time zero, depicted by the vertical dashed red line, in all perievent time histograms. Horizontal dashed red lines in z score plots indicate a z score of 3. No significant increase in MUA was seen in the CA1 pyramidal cell layer in either control or pilocarpine-treated animals. However, in both groups a significant increase in MUA, demonstrated by z-scores ≥3, was observed 5ms before and after DS.

Multiunit activity in the CA1 pyramidal cell layer showed no change in activity, as accessed by z-score, across the time period analyzed in both control and pilocarpine-treated animals, suggesting CA1 pyramidal cells were not affected by DS (Fig 4A and 4C). However, in the dentate granule cell layer in the two control animals with DS and 8 of the 9 pilocarpine-treated animals with DS, multiunit activity increased 5 ms before (Control: z-score = 3.95 ± 0.18; Pilocarpine: z-score = 4.52 ± 0.47) and 5 ms after (Control: z-score = 3.40 ± 0.40; Pilocarpine: z-score = 4.85 ± 0.56) the peak amplitude of the dentate spike, suggesting an event related increase in granule cell layer activity (Fig 4B and 4D).

## Discussion

The dentate gyrus plays an important role in gating excitatory inputs from the entorhinal cortex, and disruption of this function can result in epileptiform activity within the hippocampus. Although SE-induced changes in principal cell- and interneuron physiology have been described, little is known about the occurrence of spontaneous, large-amplitude, dentate gyrus population spikes. In this study, we used 16-channel laminar silicon probes to investigate how SE-induced changes modified large amplitude events in the CA1-DG and CA1-CA3 axis of the hippocampus in urethane anesthetized rats. In 9 of the 11 pilocarpine-treated animals tested and two control animals, we observed large amplitude events with a point of reversal and corresponding current sink in the molecular layer of the dentate gyrus similar to previously described DS [24,36]. Although there was no difference in the amplitude or width of these events between pilocarpine-treated and control animals, there was a significant increase in the frequency of DS in pilocarpine-treated animals. The increase in DS following SE suggests that dentate spike activity was also altered by the epileptogenic process. An increase in dentate spike frequency was also observed following kainic acid induced SE, suggesting that our observed increase in DS frequency was not model specific [26]. It was surprising that we did not observe interictal spikes with a site of generation in CA3, which are commonly reported following pilocarpine-induced SE [37,38]. This may be due to pyramidal cell death in CA3, the effect of urethane on network excitability [39], or a combination of both.

In previous studies, DS were observed within the hippocampus consisting of large amplitude, short duration field potentials representing synchronous discharge of dentate granule cells in both anesthetized and freely moving rats [24,25,27]. Consistent with our observations here, current source density analysis of DS revealed current sinks in the outer and middle molecular layer, suggesting these events result from population bursts in layer II stellate cells of lateral and medial entorhinal cortex, respectively [36]. However, DS with current sinks in the outer molecular layer were seen more frequently than those with sinks in the middle molecular layer [25]. Although we did not observe a difference in the frequency of events when comparing between layers, we did observe events in the outer molecular layer in more animals than in middle or inner molecular layers, suggesting lateral entorhinal input has a strong effect on DS generation following SE. Bilateral removal of the entorhinal cortex in normal rats significantly reduced the number of DS, further implicating the entorhinal perforant path projection as the main pathway for DS initiation [25].

Following SE, cell loss is observed in entorhinal cortex layers III and has been suggested to result in hyperexcitable layer II stellate cells, which project to granule cells [20,21,40]. It is possible that the increase in DS frequency is a compensatory mechanism resulting from an increase in excitatory input arriving from the entorhinal cortex since, DS have been suggested to provide a suppressive effect on the excitability of the CA3-CA1 network. Hilar interneurons increase firing during DS events while both CA1 and CA3 pyramidal cells demonstrate unaltered or suppressed firing activity [24,25]. Our observation that no ictal charges arose from the dentate granule cell layer and CA1 multiunit activity was unaffected by DS supports these findings. Urethane is also known to activate GABAergic receptors and inhibit NMDA receptors, which could limit neuronal excitability [39]. However, the observation that DS were recorded suggests the existence of synchronous population discharges in entorhinal layer II cells. It should be noted that despite urethane anesthesia, we have elicited interictal and ictal discharges with flurothyl inhalation. Importantly, Harvey and Sloviter [9] recorded 191 seizures in pilocarpine-treated rats during the chronic epileptic state and found that none of the ictal events begin in the dentate granule cells. Indeed these authors found that granule cells became progressively less excitable, rather than hyperexcitable, as mossy fiber sprouting progressed.

The presence of DS with current sinks in the inner molecular layer has not been previously reported in control animals, possibly because other investigators did not use the recording techniques employed here. Following SE mossy fibers from granule cells form excitatory recurrent collaterals onto neighboring granule cells within the inner molecular layer [16]. Hilar mossy cells, which project to basket cells in the inner molecular layer, are highly susceptible to cell death resulting in decreased excitatory input to inhibitory interneurons [13,41]. Remaining hilar mossy cells can become hyperexcitable and project directly to granule cells in the inner molecular layer [42,43]. As a result of these changes, increased excitatory input onto remaining granule cells could initiate DS within the inner molecular layer. This population of DS could represent a different class of events that are specific to the reorganized dentate gyrus and initiate activity without afferent input.

In addition to DS we also observed large amplitude spikes that displayed no phase reversal across the 16-channel laminar array. A difference in the amplitude at each contact was observed suggesting that these events were not artifacts. However, the peak amplitude of the non-reversing events was significantly smaller than both control and pilocarpine-induced DS. Given the lack of phase reversal of these events across the 16-channels, it is likely that these events were generated outside the hippocampus and traversed the recording electrode as a result of volume conduction. Note that these events occurred in the only two animals that did not display DS. The final population of events observed in the CA1-DG axis, is those with a current sink in stratum lacunosum moleculare of CA1 and therefore likely originated from the entorhinal cortex layer III [44].

It is surprising that we only observed events with electrodes in the CA1-CA3 orientation in one animal, and that these events showed no current sink in CA3 or CA1. Following a pilocarpine-induced SE insult, interictal spikes in CA3 and/or CA1 have been observed [45,46]. One explanation for this observation was profound cell death in CA3 and CA1 observed following pilocarpine-induced SE [47]. Indeed, in the animals utilized in this study, cell loss was observed in the pyramidal cell layer of CA3, while CA1 remained relatively unaltered. CA3 pyramidal cells display recurrent connections and are easily recruited to generate interictal spikes, which can propagate via the Schaffer collaterals to CA1 [48,49]. Thus, in this study the loss of these cells would decrease the probability of observing large amplitude events in both CA1 and CA3.

In conclusion, in this study we have shown that there is an increase in the frequency of DS that initiate in the molecular layer of the dentate gyrus following pilocarpine-induced SE. The majority of animals tested displayed current sinks in the outer and middle molecular layer corresponding to excitatory input from lateral and medial entorhinal cortex, respectively. Overall, the increase in frequency of these events suggests there is an increase in entorhinal input into dentate granule cells. Further, our findings support the idea that DS inhibit granule cells by activating feedforward inhibition.
