# Correction: Status Epilepticus Induced Spontaneous Dentate Gyrus Spikes: *In Vivo* Current Source Density Analysis

**DOI:** 10.1371/journal.pone.0136125

**Published:** 2015-08-14

**Authors:** Sean P. Flynn, Sylvain Barriere, Rod C. Scott, Pierre-Pascal Lenck-Santini, Gregory L. Holmes

The second author’s name is spelled incorrectly. The correct name is: Sylvain Barriere. The fourth author’s name also contains an additional space. The correct name is: Pierre-Pascal Lenck-Santini. The correct citation is: Flynn SP, Barriere S, Scott RC, Lenck-Santini P-P, Holmes GL (2015) Status Epilepticus Induced Spontaneous Dentate Gyrus Spikes: *In Vivo* Current Source Density Analysis. PLoS ONE 10(7): e0132630. doi:10.1371/journal.pone.0132630
